# Shift of the critical temperature in superconductors: a self-consistent approach

**DOI:** 10.1038/s41598-020-65790-8

**Published:** 2020-06-03

**Authors:** Alberto Cappellaro, Luca Salasnich

**Affiliations:** 1 Dipartimento di Fisica e Astronomia “Galileo Galilei”, Università di Padova, via Marzolo 8, 35131 Padova, Italy; 2 CNR-INO, via Nello Carrara, 1 - 50019 Sesto Fiorentino, Italy

**Keywords:** Statistical physics, thermodynamics and nonlinear dynamics, Physics, Condensed-matter physics, Phase transitions and critical phenomena, Superconducting properties and materials

## Abstract

Within the Ginzburg-Landau functional framework for the superconducting transition, we analyze the fluctuation-driven shift of the critical temperature. In addition to the order parameter fluctuations, we also take into account the fluctuations of the vector potential above its vacuum. We detail the approximation scheme to include the fluctuating fields contribution, based on the Hartree-Fock-Bogoliubov-Popov framework. We give explicit results for *d* = 2 and *d* = 3 spatial dimensions, in terms of easily accessible experimental parameters such as the Ginzburg-Levanyuk number Gi_(*d*)_, which is related to the width of the critical region where fluctuations cannot be neglected, and the Ginzburg-Landau parameter *κ*, defined as the ratio between the magnetic penetration length and the coherence one.

## Introduction

For over half a century, the Ginzburg-Landau (GL) theory has proven to be the most effective theoretical tool to describe the critical behaviour of physical systems in presence of symmetry breaking^1–4^. Despite its apparent simplicity, this functional approach can be successfully applied to a wide range of physical situations, such as, for instance neutral superfluids, spin systems and superconducting materials^4–6^. By relying on few well-grounded physical considerations (symmetries of the model and the analyticity of thermodynamic functions), it provides a formidable platform to understand the consequences of symmetry breaking and the role played by fluctuations close to the criticality.

Concerning conventional superconductors, it is well-known that fluctuations do not play a relevant role and the mean-field analysis of the GL theory is extremely reliable in describing the superconducting transition. This is due to the fact that the critical region cannot be accessed experimentally since it is very narrow^3^. As a consequence, certain questions, such as the order of the transition or the thermally-driven critical temperature shift, remained a purely theoretical exercise^7^ up to the first observation of high-T_*c*_ superconductivity^8–10^.

Generally, many of these high-T_*c*_ materials display a shorter coherence length *ξ*, implying a higher Ginzburg-Levanyuk number ${{\rm{Gi}}}_{(d)}$ and therefore a wider fluctuating region, where a mean-field approach is not reliable^11,12^. In particular, for a variety of materials, fluctuations appear to be the main opponent of high-T_*c*_ superconductivity. Since the critical temperature shift is a non-universal property of physical systems, it is important to properly understand how they affect, and eventually destroy, the superconducting phase^13,14^. For instance, in multiband superconductors it has been found that fluctuations can be suppressed by switching on a Josephson-like coupling between the bands^15^.

In this paper we consider the minimal coupling of the usual ${\psi }^{4}$-theory with the electromagnetic field. We aim to compute the fluctuation-driven shift of the critical temperature (compared to the mean-field scheme) by taking into account both the order parameter and the vector potential fluctuations. In order to perform this task we make use of an improved saddle-point equation, where fluctuations are taken into account within the so-called Hartree-Fock-Bogoliubov-Popov scheme. This approximation scheme is one of the many field-theoretical strategies to model finite-temperature degenerate gases (for a review see^16^), where it has been proven to provide a reliable picture for bosonic alkali vapors placed in an external confinement^17–19^. Here, this approach is applied to the Ginzburg-Landau equations, reading a modified saddle-point equation where additional terms are present, depending on the average of the square modulus of fluctuations. Once one has a strategy to self-consistently compute these quantities, the critical temperature shift can be easily derived.

The paper is structured as follows: first, we review some general features of the Ginzburg-Landau theory. In particular, we introduce the minimal coupling with the electromagnetic field. Then we present our strategy to include the fluctuations in an improved saddle-point (or Ginzburg-Landau) equation. We give explicit results for $d=2$ and $d=3$ dimensions, where the shift turns out to depend only on the Ginzburg-Levanyuk number ${{\rm{Gi}}}_{(d)}$ and the parameter $\kappa$, the latter being the ratio between the magnetic penetration length and the coherence one. Our analytical formulas for the shift of the critical temperature, directly obtained from the Ginzburg-Landau functional, are a generalization of familiar ones^20^, which take into account only the fluctuations of the order parameter.

## Results

### The Ginzburg-Landau formulation: general features

Concerning the normal-to-superconductor transition, close to the critical temperature, the free energy of a single-band superconductor can be split into

1 $$ {\mathcal F} ={ {\mathcal F} }_{s}+{ {\mathcal F} }_{n},$$

with ${ {\mathcal F} }_{n}$ the contribution of the disordered (i.e. normal) component, while ${ {\mathcal F} }_{s}$ models the contribution due to the emergence of an ordered phase (i.e. superconductor) characterized by an order parameter $\psi ({\bf{r}})$ acquiring a non-zero value below a certain critical temperature to determine. The great intuition of Landau and Ginzburg consisted in writing down the latter in terms of few simple terms based on the symmetries to which the theory obeys. In our case, this implies^20^

2 $$\begin{array}{l}{ {\mathcal F} }_{s}[\psi ,{\bf{A}}]={\int }_{V}\,{d}^{d}{\bf{r}}\,\left[a(T)|\psi {|}^{2}+\frac{b}{2}|\psi {|}^{4}+\gamma {D}_{\mu }\psi \,{D}_{\mu }^{\ast }{\psi }^{\ast }+\frac{{(\nabla \wedge {\bf{A}})}^{2}}{2{\mu }_{0}}\right]\end{array}$$

where

3 $${D}_{\mu }={\partial }_{\mu }-i{e}^{\ast }{A}_{\mu }({\bf{r}})$$

is the gauge-invariant derivative coupling the order parameter $\psi ({\bf{r}})$ with the vector potential ${\bf{A}}({\bf{r}})$, with e* being the effective charge. In the equations above, the index $\mu$ labels the component of the vector ${\bf{A}}$. One has also to recall that the order parameter $\psi$ has to be assumed as a complex field if we aim to describe the normal-to-superconducting transition.

Concerning the couplings of the theory described by ${ {\mathcal F} }_{s}$ in Eq. (2), $b$ and $\gamma$ can be taken as positive constants, while the coefficient of the $|\psi {|}^{2}$-term contains the temperature dependence. Simple considerations lead to the well-known conclusion that this dependence is linear, namely

4 $$a(T)=\alpha {k}_{B}(T-{T}_{c0})$$

with $\alpha  > 0$ and ${k}_{B}$ the Boltzmann constant. In the equation above, ${T}_{c0}$ is the mean-field critical temperature.

It has to be remarked that Eq. (2) holds in absence of an external magnetic field. In order to model this situation, ${ {\mathcal F} }_{s}[\psi ;{\bf{A}}]$ must be modified adding a term proportional to $(\nabla \wedge A)\cdot {\bf{H}}$. However, in this paper we limit our analysis to case of no applied external field.

It is immediate to show that, with the ansatz $\psi ({\bf{r}})={\psi }_{0}$ and ${\bf{A}}({\bf{r}})={\bf{0}}$, one can find the usual saddle-point solution

5 $$|{\psi }_{0}^{({\rm{mf}})}{|}^{2}=\{\begin{array}{ll}0 & {\rm{for}}\,a({\rm{T}}) > 0\\ -\frac{a(T)}{b} & {\rm{for}}\,a({\rm{T}}) < 0\end{array}\mathrm{}.$$

Thus, the order parameter acquires a non-zero value when $a(T)$ changes its sign. Within the mean-field scheme, the phase transition then occurs at a temperature given by the solution of

6 $$a({T}_{c0})=0.$$

This simple approach lacks every information about the fluctuations of the order parameter and the vector potential. For conventional superconductors, the critical region where fluctuations are crucial is very small and cannot be observed.

However, with the discovery of novel materials displaying exotic superconductivity, it appears clear that deviations from the mean-field picture have to be explored very carefully.

### Including the fluctuations: the HFBP scheme

#### The saddle-point equation in the HFBP approximation

Differently from the case of neutral superfluids, the Ginzburg-Landau functional for a superconducting system, as given by Eq. (2), depends on two different fields because of the minimal coupling with the electromagnetic field.

Thus, the saddle-point configuration of the system has to be determined by searching for the stationary trajectories of ${ {\mathcal F} }_{s}[\psi ,{\bf{A}}]$. In other words, one has to solve the system

7 $$\frac{\delta { {\mathcal F} }_{s}}{\delta {\psi }^{\ast }}=0\,{\rm{and}}\,\frac{\delta { {\mathcal F} }_{s}}{\delta {\bf{A}}}=0$$

where *δ*/*δ*(•) has to be intended in the sense of first variation. The equation resulting from the variation with respect to ${\psi }^{\ast }$ reads

8 $$[a(T)+b|\psi {|}^{2}-\gamma {\nabla }^{2}+\gamma {({e}^{\ast })}^{2}|{\bf{A}}{|}^{2}+2i\gamma {e}^{\ast }{\bf{A}}\cdot \nabla ]\psi =\mathrm{0,}$$

while $\delta { {\mathcal F} }_{s}/\delta {\bf{A}}=0$ leads us to the following equation

9 $$\left[2\gamma {({e}^{\ast })}^{2}|\psi {|}^{2}+\frac{\nabla \wedge \nabla \wedge }{{\mu }_{0}}\right]{\bf{A}}=i{e}^{\ast }(\psi \nabla {\psi }^{\ast }-{\psi }^{\ast }\nabla \psi ).$$

In both Eqs. (8) and (9) the spatial dependence of both fields is left intended. Obviously, assuming the ansatz $\psi ({\bf{r}})={\psi }_{0}$ and ${\bf{A}}={\bf{0}}$ as in the previous section, the Ginzburg-Landau equation are solved by Eq. (5). It is then clear that a saddle-point configuration exists for a uniform order parameter and in absence of a vector potential.

In order to encode the thermal fluctuations in a saddle-point scheme, let us then split the order parameter

10 $$\psi ({\bf{r}})={\psi }_{0}+\eta ({\bf{r}})$$

where ${\psi }_{0}$ is a constant which can be assumed as real but it is not necessarily given by ${\psi }_{0}^{({\rm{mf}})}$ in Eq. (5). On the other hand, $\eta ({\bf{r}})$ is the space dependent fluctuation field. The crucial feature concerning $\psi ({\bf{r}})$ as defined above is that

11 $${\psi }_{0}\equiv \langle \psi ({\bf{r}})\rangle $$

and, as an immediate consequence,

12 $$\langle \eta \rangle =\langle {\eta }^{\ast }\rangle =0.$$

The thermal average $\langle \ldots \rangle$ has to be intended as performed over a proper statistical ensemble where a global gauge symmetry can be spontaneously broken.

For the vector potential ${\bf{A}}({\bf{r}})$, we only consider fluctuations ${\mathscr{A}}({\bf{r}})$ above its vacuum **0**, namely

13 $${\bf{A}}({\bf{r}})={\bf{0}}+{\mathscr{A}}({\bf{r}}).$$

Now, we proceed by replacing Eq. (10) in Eq. (8), such that

14 $$\begin{array}{c}a(T){\psi }_{0}+a(T)\eta +b{\psi }_{0}^{3}+b{\psi }_{0}^{2}(2\eta +{\eta }^{\ast })+b{\psi }_{0}(2|\eta {|}^{2}+{\eta }^{2})+b|\eta {|}^{2}\eta \\ \,-\,\gamma {\nabla }^{2}\eta +\gamma {({e}^{\ast })}^{2}|{\mathscr{A}}{|}^{2}{\psi }_{0}+\gamma {({e}^{\ast })}^{2}|{\mathscr{A}}{|}^{2}\eta +2i\gamma {e}^{\ast }{\mathscr{A}}\cdot \nabla \eta =0.\end{array}$$

We are interested in how fluctuations modify the uniform background ${\psi }_{0}$ within the broken symmetry phase, i.e. for $T < {T}_{c}$. We remark that, according to this framework, the critical temperature ${T}_{c}\ne {T}_{c0}$, since Eq. (6) does not take into account the role of fluctuations.

In order to derive an equation for ${\psi }_{0}$ including a contribution due to fluctuations, both of the order parameter and the vector potential, we take the thermal average of Eq. (14). Because of Eq. (12), linear terms in $\eta$ and ${\eta }^{\ast }$ are erased by default. One is then left with

15 $$\begin{array}{l}\begin{array}{c}[a(T)+2b\langle |\eta {|}^{2}\rangle +b\langle {\eta }^{2}\rangle +\gamma {({e}^{\ast })}^{2}\langle |{\mathscr{A}}{|}^{2}\rangle ]\,{\psi }_{0}+b{\psi }_{0}^{3}+b\langle |\eta {|}^{2}\eta \rangle \\ \,+\,\gamma {({e}^{\ast })}^{2}\langle |{\mathscr{A}}{|}^{2}\eta \rangle +2i\gamma {e}^{\ast }\langle {\mathscr{A}}\cdot \nabla \eta \rangle =0,\end{array}\end{array}$$

which still appears rather complicated. Up to this point, we still have not fixed the gauge for the vector potential. It is a well-known fact that the most natural choice is the Coulomb (or transverse) gauge, where

16 $$\nabla \cdot {\mathscr{A}}=0.$$

Actually, besides the convenience matter, this fixing has profound physical consequences. Indeed, it has been shown that only with Eq. (16) the order parameter correlations $\langle \psi ({\bf{r}})\psi \mathrm{(0)}\rangle$ acquires a long-ranged character (see, for instance^5^). Concerning Eq. (15), let us notice that, through a Fourier transformation, the Coulomb gauge is equivalently given by $\tilde{{\mathscr{A}}}({\bf{q}})\perp {\bf{q}}$, with **q** a wavevector in the reciprocal space. This implies that $\langle {\mathscr{A}}\cdot \nabla \eta \rangle =0$.

Moving further, we simplify Eq. (15) by means of the following approximation scheme. First, we neglect the three-field correlations, namely $\langle |\eta {|}^{2}\eta \rangle \simeq 0$ and $\langle |{\mathscr{A}}{|}^{2}\eta \rangle \simeq 0$. By drawing an analogy with the analysis of bosonic gases at finite temperatures, this corresponds to the Hartree-Fock-Bogoliubov (HFB) scheme^17^. Moreover, we also discard the anomalous average, namely $\langle {\eta }^{2}\rangle =\langle {({\eta }^{\ast })}^{2}\rangle \simeq 0$, according to the so-called Popov approximation of the HFB framework (HFBP in the following).

Thus, Eq. (15) finally reads

17 $$[a(T)+2b\langle |\eta {|}^{2}\rangle +\gamma {({e}^{\ast })}^{2}\langle |{\mathscr{A}}{|}^{2}\rangle ]\,{\psi }_{0}+b{\psi }_{0}^{3}=0.$$

whose solution, for $T < {T}_{c}$, is given by

18 $${\psi }_{0}^{2}=-\,\frac{a(T)+2b\langle |\eta {|}^{2}\rangle +\gamma {({e}^{\ast })}^{2}\langle |{\mathscr{A}}{|}^{2}\rangle }{b}.$$

From the equation is then immediate to derive the generalization of Eq. (6) for the critical temperature, i.e.

19 $$a({T}_{c})+2b{\langle |\eta {|}^{2}\rangle }_{c}+\gamma {({e}^{\ast })}^{2}{\langle |{\mathscr{A}}{|}^{2}\rangle }_{c}=0$$

where ${\langle \ldots \rangle }_{c}$ implies that the average has to be computed at the critical point.

In contrast with Eq. (6), Eq. (19) takes into account the presence of fluctuations both in the order parameter and the vector potential, through the averages $\langle |\eta {|}^{2}\rangle$ and $\langle |{\mathscr{A}}{|}^{2}\rangle$.

#### Averages in the HFBP scheme

The shift of the critical temperature as in Eq. (19) requires the calculation of $\langle |\eta {|}^{2}\rangle$ and $\langle |{\mathscr{A}}{|}^{2}\rangle$ at the criticality. As detailed in the Methods section, they can be computed by taking the GL equations for the fluctuating fields $\eta ({\bf{r}})$ and ${\mathscr{A}}({\bf{r}})$ as starting point (cfr. Eqs. (50) and (54)). From there, as outlined below, one can infer the corresponding Gaussian functional (i.e. a free energy, similarly to Eq. (2)) driving the thermodynamic properties of fluctuations. Indeed both Eqs. (50) and (54) in Methods are the first variation of a Gaussian functional. By working slightly above the critical temperature (i.e. $T\to {T}_{c}^{+}$), where ${\psi }_{0}=0$, Eq. (50) reads

20 $$[a(T)+2b\langle |\eta {|}^{2}\rangle +\gamma {({e}^{\ast })}^{2}\langle |{\mathscr{A}}{|}^{2}\rangle -\gamma {\nabla }^{2}]\eta =0,$$

which is the trajectory stationarizing the functional

21 $${ {\mathcal F} }_{\eta }^{(g)}[\eta ,{\eta }^{\ast }]={\int }_{V}\,{d}^{d}{\bf{r}}\,{\eta }^{\ast }[a(T)+2b\langle |\eta {|}^{2}\rangle +\gamma {({e}^{\ast })}^{2}\langle |{\mathscr{A}}{|}^{2}\rangle -\gamma {\nabla }^{2}]\eta $$

with $V$ being the large *d*-dimensional volume enclosing the system.

Similarly, from Eq. (54), one can infer the corresponding functional for ${\mathscr{A}}$, i.e.

22 $${ {\mathcal F} }_{{\mathscr{A}}}^{(g)}[{\mathscr{A}}]={\int }_{V}\,{d}^{d}{\bf{r}}\,\left[\gamma {({e}^{\ast })}^{2}\langle |\eta {|}^{2}\rangle |{\mathscr{A}}{|}^{2}+\frac{{(\nabla \wedge {\mathscr{A}})}^{2}}{2{\mu }_{0}}\mathrm{}.\right]$$

Each one of ${ {\mathcal F} }_{\eta }^{(g)}[\eta ]$ and ${ {\mathcal F} }_{{\mathscr{A}}}^{(g)}[{\mathscr{A}}]$ are related to their corresponding partition function, from which it is usually easy to compute average values and correlations, since both of them are (functional) Gaussian integrals.

The presence of differential operators suggests that the calculation is easier in the Fourier space. Thus, with

23 $$\eta ({\bf{r}})=\frac{1}{\sqrt{V}}\,\sum _{{\bf{q}}}\,{e}^{i{\bf{q}}\cdot {\bf{r}}}\,\tilde{\eta }({\bf{q}})$$

and

24 $${\mathscr{A}}({\bf{r}})=\frac{1}{\sqrt{V}}\,\sum _{{\bf{q}}}\,{e}^{i{\bf{q}}\cdot {\bf{r}}}\,\tilde{{\mathscr{A}}}({\bf{q}})$$

Equations (21) and (22) transform as, respectively,

25 $${ {\mathcal F} }_{\eta }^{(g)}=\sum _{{\bf{q}}}\,[a(T)+2b\langle |\eta {|}^{2}\rangle +\gamma {({e}^{\ast })}^{2}\langle |{\mathscr{A}}{|}^{2}\rangle +\gamma {q}^{2}]|\tilde{\eta }({\bf{q}}{)|}^{2}$$

plus

26 $${ {\mathcal F} }_{{\mathscr{A}}}^{(g)}=\sum _{{\bf{q}}}\,\left[\gamma {({e}^{\ast })}^{2}\langle |\eta {|}^{2}\rangle +\frac{{q}^{2}}{2{\mu }_{0}}\right]|\tilde{{\mathscr{A}}}({\bf{q}}{)|}^{2}\mathrm{}.$$

Now, since both ${ {\mathcal F} }_{\eta }^{(g)}[\tilde{\eta }]$ and ${ {\mathcal F} }_{{\mathscr{A}}}^{(g)}[\tilde{{\mathscr{A}}}]$ are Gaussian, it is immediate to infer that

27 $$\langle |\eta {|}^{2}\rangle =\frac{1}{\beta V}\,\sum _{{\bf{q}}}\,\frac{1}{a(T)+2b\langle |\eta {|}^{2}\rangle +\gamma {({e}^{\ast })}^{2}\langle |{\mathscr{A}}{|}^{2}\rangle +\gamma {q}^{2}}.$$

At the criticality Eq. (19) holds, therefore the equation above is simplified into

28 $${\langle |\eta {|}^{2}\rangle }_{c}=\frac{{k}_{B}{T}_{c}}{V}\,\sum _{{\bf{q}}}\,\left(\frac{1}{\gamma {q}^{2}}\right)\mathrm{}.$$

Let us note that this result is the same one can derive in absence of the minimal coupling with the electromagnetic field (i.e. ${\bf{A}}={\bf{0}}$). The crucial point is the fact that, on the contrary, ${\langle |{\mathscr{A}}{|}^{2}\rangle }_{c}$ is affected by the fluctuation of the order parameter. Indeed, from Eq. (26), one gets

29 $${\langle |{\mathscr{A}}{|}^{2}\rangle }_{c}=\frac{{k}_{B}{T}_{c}}{V}\,\sum _{{\bf{q}}}\,\frac{d-1}{2\gamma {({e}^{\ast })}^{2}{\langle |\eta {|}^{2}\rangle }_{c}+{q}^{2}/{\mu }_{0}}$$

where the factor $(d-\mathrm{1)}$ is a consequence of the Coulomb gauge, setting to zero the component of $\tilde{{\mathscr{A}}}({\bf{q}})$ parallel to ${\bf{q}}$. The vector potential then has only $(d-\mathrm{1)}$ non-zero transverse components.

It worth to remember, at this point, the main results we have obtained by means of the HFBP approximation scheme. First, we have derived the equation describing the shift of the critical temperature (compared to the usual saddle-point result in Eq. (6)). In order to actually solve Eq. (19), we also need the average values (at the criticality) ${\langle |\eta {|}^{2}\rangle }_{c}$ and ${\langle |{\mathscr{A}}{|}^{2}\rangle }_{c}$, respectively in Eqs. (28) and (29).

In the following, we are going to consider the continuum limit, namely (in spherical coordinates)

30 $$\sum _{{\bf{q}}}\to \frac{V}{{\mathrm{(2}\pi )}^{d}}{S}_{d}\,{\int }_{{q}_{0}}^{\Lambda }\,dq\,{q}^{d-1},$$

with ${S}_{d}=2{\pi }^{d/2}/\Gamma (d/2)$ is the whole solid *d*-dimensional solid angle. In the equation above, we have introduced both an ultraviolet and an infrared cutoff to keep eventual divergences under control.

In the following section, we consider the case of $d=2$ and $d=3$ spatial dimensions. An extremely interesting problem is represented by the dimensional crossover, i.e. the analysis of a thin film but with a finite thickness *δ*. This physical realization has been investigated in presence of a disordered environment^21–23^, reading an additional (logarithmic) shift of the critical temperature depending on *δ* and a properly defined diffusion coefficient.

### Fluctuation-driven critical temperature shift

#### **The case***d* = 2

In $d=2$, by taking the continuum limit as in Eq. (30), Eqs. (28) and (29) easily lead us to

31 $${\langle |\eta {|}^{2}\rangle }_{c}=\frac{{k}_{B}{T}_{c}}{2\pi \gamma }\,\mathrm{ln}\,\left(\frac{\Lambda }{{q}_{0}}\right)$$

and

32 $${\langle |{\mathscr{A}}{|}^{2}\rangle }_{c}=\frac{{\mu }_{0}{k}_{B}{T}_{c}}{4\pi }\,\mathrm{ln}\,\left(\frac{{\Lambda }^{2}+2{\mu }_{0}\gamma {({e}^{\ast })}^{2}{\langle |\eta {|}^{2}\rangle }_{c}}{{q}_{0}^{2}+2{\mu }_{0}\gamma {({e}^{\ast })}^{2}{\langle |\eta {|}^{2}\rangle }_{c}}\right)\mathrm{;}.$$

According to^20^, a reasonable choice for the UV cutoff is $\Lambda \simeq \mathrm{1/}{\xi }_{c}$, with

33 $${\xi }_{c}=\sqrt{\frac{\gamma }{\alpha {k}_{B}{T}_{c}}}.$$

Concerning the infrared cutoff ${q}_{0}$, we define it in terms of the Ginzburg-Levanyuk number through

34 $${q}_{0}=\sqrt{\frac{\alpha {k}_{B}{T}_{c}}{\gamma }{{\rm{Gi}}}_{(2)}}\mathrm{}.$$

The definition of the Ginzburg-Levanyuk number ${{\rm{Gi}}}_{(d)}$ strongly depends on the system dimensionality. Again, according to^20^, in $d=2$ one has

35 $${{\rm{Gi}}}_{(2)}=\frac{b}{4\pi \gamma \alpha }.$$

**Figure 1:**
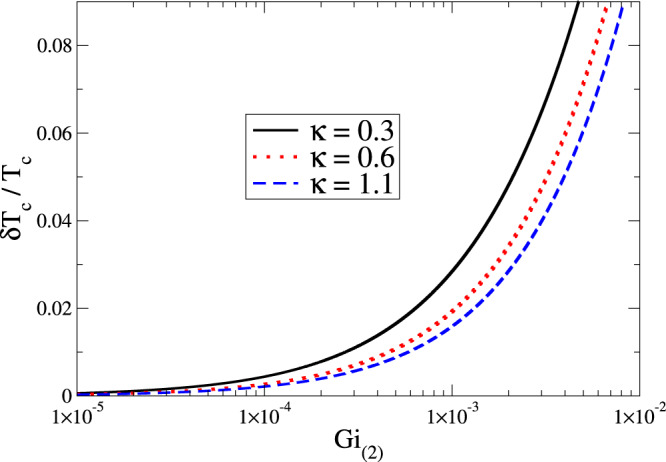
Shift of the critical temperature in two spatial dimensions according to Eq. (40) as a function of Gi_(2)_ for three different values of $\kappa$. In the panel above, $\delta {T}_{c}={T}_{c0}-{T}_{c}$.

The UV cutoff is naturally defined by Eq. (33), which specifies the minimal size of spatial fluctuations. The choice for the infrared cutoff in Eq. (34) is less obvious and has to rely upon a renormalization-group (RG) argument^15^. Within the Wilson’s standard framework of momentum-shell integration^4,5^, the flow equation for cutoff-dependent ${a}_{{\lambda }}(T)$ can be simplified by assuming that the parameter $b$ does not flow (i.e. it is equal to its bare value). The cutoff *λ*, separating the slow from fast modes in Wilson’s approach, has an upper (i.e. ultraviolet) limit equal to ${{\lambda }}_{\infty }\to \Lambda =1/{\xi }_{c}$ with ${\xi }_{c}$ as in Eq. (33). In $d=2$, this approximation the equation for ${a}_{{\lambda }}(T)$ reads a logarithmic solution diverging when ${\lambda }\to 0$, naturally introducing the infrared cutoff ${q}_{0}$ as in Eq. (34). By solving simultaneously the RG equations for ${a}_{{\lambda }}$ and ${b}_{{\lambda }}$ the divergence disappears but, remarkably, it has been shown that the logarithmic approximation outlined above works really well for ${{\rm{Gi}}}_{(2)}\ll 1/2$, which is exactly the range of values explored in this paper (cfr. Fig. 1).

Therefore, by following the cutoff prescriptions outlined above, Eqs. (31) and (32) result in

36 $${\langle |\eta {|}^{2}\rangle }_{c}=\frac{{k}_{B}{T}_{c}}{4\pi \gamma }\,\mathrm{ln}\left(\frac{1}{{{\rm{Gi}}}_{(2)}}\right)$$

and

37 $${\langle |{\mathscr{A}}{|}^{2}\rangle }_{c}=\frac{{\mu }_{0}{k}_{B}{T}_{c}}{4\pi }\,\mathrm{ln}\left[\frac{1+\frac{{{\rm{Gi}}}_{(2)}}{{\kappa }^{2}}\,\mathrm{ln}\left(\frac{1}{{{\rm{Gi}}}_{(2)}}\right)}{{{\rm{Gi}}}_{(2)}+\frac{{{\rm{Gi}}}_{(2)}}{{\kappa }^{2}}\,\mathrm{ln}\left(\frac{1}{{{\rm{Gi}}}_{(2)}}\right)}\right]\mathrm{}.$$

The parameter $\kappa$ is the usual ratio between the magnetic penetration length ${\lambda }(T)$ and the coherence length $\xi (T)$, i.e. $\kappa ={\lambda }(T)/\xi (T)$. We recall that $\kappa$ is a crucial quantity in the GL theory for superconductors, since type-I superconductors all have $\kappa  < 1/\sqrt{2}$. On the contrary, the condition $\kappa  > 1/\sqrt{2}$ characterizes type-II superconducting materials. By assuming

38 $$\xi (T)=\sqrt{\frac{\gamma }{a(T)}}\,{\rm{and}}\,{\lambda }(T)=\sqrt{\frac{b}{2{\mu }_{0}{({e}^{\ast })}^{2}\gamma a(T)}},$$

one easily gets

39 $$\kappa =\frac{{\lambda }(T)}{\xi (T)}=\sqrt{\frac{b}{2{\mu }_{0}{({e}^{\ast })}^{2}{\gamma }^{2}}}\mathrm{}.$$

It has to be remarked that the equation above still holds for $d=3$.

By replacing Eqs. (36) and (37) in Eq. (19), the shift of the critical temperature in $d=2$, compared to the usual saddle-point picture, is given by

40 $$\frac{{T}_{c0}-{T}_{c}}{{T}_{c}}=2{{\rm{Gi}}}_{\mathrm{(2)}}\,\mathrm{ln}\left(\frac{1}{{{\rm{Gi}}}_{(2)}}\right)+\frac{{{\rm{Gi}}}_{(2)}}{2{\kappa }^{2}}\,\mathrm{ln}\left[\frac{1+\frac{{{\rm{Gi}}}_{(2)}}{{\kappa }^{2}}\,\mathrm{ln}\left(\frac{1}{{{\rm{Gi}}}_{(2)}}\right)}{{{\rm{Gi}}}_{(2)}+\frac{{{\rm{Gi}}}_{(2)}}{{\kappa }^{2}}\,\mathrm{ln}\left(\frac{1}{{{\rm{Gi}}}_{(2)}}\right)}\right]\mathrm{}.$$

We stress that only two parameters, the Ginzburg-Levanyuk number ${{\rm{Gi}}}_{(2)}$ and the Ginzburg-Landau ratio $\kappa$, drive the shift of the critical temperature in two spatial dimensions. Equation (40) is one of the main results of the paper. The first addend of this equation, already known in literature^15,20^, takes into account the thermal fluctuations of the order parameter. The second addend takes instead into account the thermal fluctuations of the electromagnetic vector potential around its vacuum. In Fig. 1 we report the behaviour of the critical temperature shift given by Eq. (40) (with $\delta {T}_{c}={T}_{c0}-{T}_{c}$) as a function of ${{\rm{Gi}}}_{(2)}$ for three different values of $\kappa$. Let us remark that the displacement from the mean-field prediction ${T}_{c0}$ is progressively reduced by moving towards to the type-II regime.

It is important to recall that two-dimensional physical systems require from us to also consider the eventual occurring of the Berezinskii-Kosterlitz-Thouless (BKT) transition^24–26^. Its peculiar behaviour can be understood in terms of phase fluctuations of the order parameter. Indeed, a complex field can be naturally characterized in terms of two real fields by means of the phase-amplitude representation

41 $$\psi ({\bf{r}})={\Psi }_{0}\,\exp \,\{i\phi ({\bf{r}}\mathrm{)\}}.$$

In the equation above, ${\Psi }_{0}$ can be taken as the uniform solution of the GL equations. By neglecting the crucial role played by the phase field $\phi ({\bf{r}})$, the amplitude acquires a non-zero value for $T < {T}_{c}$, with ${T}_{c}$ being the shifted (compared to the mean-field result) critical temperature in Eq. (40). On the other hand, the phase field $\phi ({\bf{r}})$ is obviously defined on a compact support. As a consequence, the system can display nontrivial topological excitations in form of quantized vortices.

In Methods we outline a procedure providing, in first approximation, the additional BKT shift^15,20^ by means of the Nelson-Kosterlitz criterion^27^. According to this approach, ${T}_{BKT}$ is shifted with respect to the unrenormalized contribution in Eq. (40) by

42 $$\frac{{T}_{c}-{T}_{BKT}}{{T}_{BKT}}=4\,{{\rm{Gi}}}_{(2)}\mathrm{}.$$

It is important to remark that, while the Eq. (42) has a very simple appearance, the fundamental input is the critical temperature computed in Eq. (40).

#### **The case***d* = 3

In the case $d=3$, the integration over $q$ as prescribed by Eq. (30) of Eqs. (28) and (29) gives us back

43 $${\langle |\eta {|}^{2}\rangle }_{c}=\frac{{k}_{B}{T}_{c}\Lambda }{2{\pi }^{2}\gamma }$$

and

44 $${\langle |{\mathscr{A}}{|}^{2}\rangle }_{c}=\frac{{\mu }_{0}{k}_{B}{T}_{c}}{{\pi }^{2}}\left[\Lambda -\sqrt{2{\mu }_{0}{({e}^{\ast })}^{2}\gamma {\langle |\eta {|}^{2}\rangle }_{c}}\,\arctan \,\left(\frac{\Lambda }{\sqrt{2{\mu }_{0}{({e}^{\ast })}^{2}\gamma {\langle |\eta {|}^{2}\rangle }_{c}}}\right)\right]\mathrm{}.$$

No infrared divergence arises by performing the integration leading to the equations above, so we have safely taken the limit ${q}_{0}\to 0$. We also approximate the $\arctan (\ldots )\to \pi /2$ since it provides only a subleading contribution, compared to the coefficient in front of it. However, an ultraviolet divergence are still present: as for $d=2$, we assume $\Lambda \simeq 1/{\xi }_{c}$, with ${\xi }_{c}$ given by Eq. (33), holding also in three spatial dimensions. The Ginzburg-Levanyuk number, on the contrary, reads^20^

45 $${{\rm{Gi}}}_{(3)}=\frac{{b}^{2}}{64{\pi }^{2}\alpha {\gamma }^{3}}{k}_{B}{T}_{c}\mathrm{}.$$

Consequently,

46 $${\langle |\eta {|}^{2}\rangle }_{c}=\frac{4\alpha {k}_{B}{T}_{c}}{\pi b}\sqrt{{{\rm{Gi}}}_{(3)}}$$

and

47 $$\begin{array}{l}{\langle |{\mathscr{A}}{|}^{2}\rangle }_{c}=\frac{{\mu }_{0}{k}_{B}{T}_{c}}{{\pi }^{2}}\left[\sqrt{\alpha {k}_{B}{T}_{c}}\gamma -\sqrt{2\pi {\mu }_{0}{({e}^{\ast })}^{2}\alpha \gamma \sqrt{{{\rm{Gi}}}_{(3)}}\frac{{k}_{B}{T}_{c}}{b}}\right]\mathrm{}.\end{array}$$

**Figure 2:**
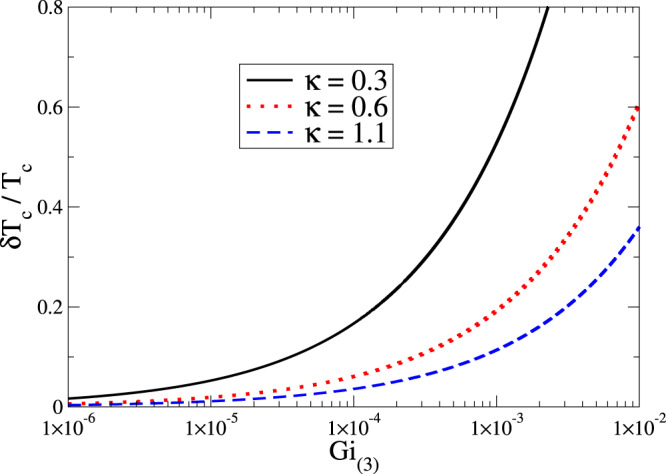
Shift of the critical temperature in three spatial dimensions according to Eq. (48) as a function of Gi_(3)_ for different values of $\kappa$. For the sake of comparison we have made use of the same values of Fig. 1. In the panel above, $\delta {T}_{c}={T}_{c0}-{T}_{c}$.

By inserting Eqs. (46) and (47) in Eq. (19), up to the leading term in ${{\rm{Gi}}}_{(3)}$, the shift of the critical temperature results in

48 $$\begin{array}{l}\frac{{T}_{c0}-{T}_{c}}{{T}_{c}}=\frac{8}{\pi }\sqrt{{{\rm{Gi}}}_{(3)}}+\frac{4}{\pi {\kappa }^{2}}\sqrt{{{\rm{Gi}}}_{(3)}}\end{array}$$

with $\kappa$ defined in Eq. (39). Similarly to the $d=2$ case, in Fig. 2 we plot Eq. (48) as a function of the Ginzburg-Levanyuk number for different values of the ratio $\kappa$. Again, we see that, at higher values of $\kappa$, namely for higher penetration lengths or lower coherence ones, $({T}_{c0}-{T}_{c})/{T}_{c}$ is remarkably reduced.

Again, by assuming the minimal coupling with the electromagnetic field, the Ginzburg-Levanyuk number ${{\rm{Gi}}}_{(3)}$ and the Ginzburg-Landau ratio $\kappa$ are all one needs to characterize the critical temperature shift from the usual mean-field scheme. Equation (48) is the another important result of our paper. The first addend of this equation is already known in literature^4,20^ while the second addend, related to the fluctuations of the vector potential, is instead a novel result.

## Discussion

In this paper, we have presented a self-consistent approach to include the fluctuations of order parameter and vector potential in an improved saddle-point equation. As a consequence, it has been possible to derive the fluctuation-driven shift of ${T}_{c}$ both in $d=3$ and $d=2$. Remarkably, this displacement from the usual mean-field results only depends from two combinations of the GL coupling constants: the Ginzburg-Levanyuk number ${{\rm{Gi}}}_{(d)}$, related to the width of the critical region, and the ratio $\kappa ={\lambda }(T)/\xi (T)$, dividing type-I from type-II superconductors.

In presence of a wide critical region, namely a high value of ${{\rm{Gi}}}_{(d)}$, it is crucial to properly consider not only the role of fluctuations on the various thermodynamic functions, but also the eventual strategies apt to control them. This is not a purely academical question; on the contrary it can have relevant technological applications, since this kind of materials are the ones displaying pairing mechanisms leading to high-temperature superconductivity, such as electron-hole superfluidity^28–30^. Indeed, as already mentioned, an inter-band Josephson-like coupling has been proved to contain the detrimental effect of fluctuations and preserve an optimal superconductivity regime^15^, also in reduced dimensionality, where coherent behaviour is otherwise greatly suppressed^31^.

By adopting this point of view, in this paper we have considered a single-band structure but in presence of a minimal coupling to the electromagnetic field. As a consequence, it turns out the fluctuations of the vector potential ${\mathscr{A}}$ that the critical temperature acquires an additional shift depending from $\kappa$ (cfr. Eqs. (40) and (48)). In other words, it depends on how much the material sample can be penetrated by the magnetic fields. A natural extension of this analysis is involving the presence of at least another band with a proper coupling, together with the possibility to introduce the particle statistics moving to a quantum framework which is closer, for instance, to the phenomenology of the BCS-BEC crossover^13,32^.

## Methods

### The GL equations for the fluctuating fields

In this section we detail the derivation of the GL equations for the fluctuations of the order parameter and the vector potential, namely $\eta ({\bf{r}})$ and ${\mathscr{A}}({\bf{r}})$. These equations then serve as the starting point to engineer a (Gaussian) functional accounting for the statistical properties of the fluctuating fields. This task can be fulfilled by looking back at Eq. (14). Here, the HFB scheme prescribes that all the terms more than linear in the fluctuating fields are decoupled with a couple-by-couple average. For instance, $|\eta {|}^{2}\eta \simeq 2\langle |\eta {|}^{2}\rangle \eta +\langle {\eta }^{2}\rangle {\eta }^{\ast }$, $|\eta {|}^{2}\simeq \langle |\eta {|}^{2}\rangle$ and $|{\mathscr{A}}{|}^{2}\eta \simeq \langle |{\mathscr{A}}|\rangle \eta +2\langle \eta {\mathscr{A}}\rangle \cdot {\mathscr{A}}$. In addition, the Popov prescription imposes $\langle {\eta }^{2}\rangle \simeq 0$. We also assume that the order parameter and the vector potential fluctuates independently, so

49 $$\langle \eta \,\delta {A}_{\mu }\rangle =\langle \eta \rangle \langle \delta {A}_{\mu }\rangle =0$$

where the last equality is due to Eq. (12). This series of considerations leads us to the GL equation for the order parameter fluctuations,

50 $$[a(T)+2b\langle |\eta {|}^{2}\rangle +\gamma {({e}^{\ast })}^{2}\langle |{\mathscr{A}}{|}^{2}\rangle \eta +2b{\psi }_{0}^{2}-\gamma {\nabla }^{2}]+b{\psi }_{0}^{2}{\eta }^{\ast }=0.$$

The corresponding equation for ${\mathscr{A}}$ can be easily derived by following the same steps detailed above, together with the Coulomb gauge. More in detail, by replacing Eqs. (10) and (13) in (9), together with the assumption in Eq. (49), this procedure transforms the left-hand-side (LHS) as

51 $$\left[2\gamma {({e}^{\ast })}^{2}|\psi {|}^{2}+\frac{\nabla \wedge \nabla \wedge }{{\mu }_{0}}\right]{\bf{A}}\simeq 2\gamma {({e}^{\ast })}^{2}({\psi }_{0}^{2}+\langle |\eta {|}^{2}\rangle ){\mathscr{A}}+\frac{\nabla \wedge \nabla \wedge {\mathscr{A}}}{{\mu }_{0}}\mathrm{}.$$

Concerning the right-hand-side of Eq. (9), additional comments are in order. First, let us note that, because of Eq. (10), terms linear in $\nabla \eta$ and $\nabla \eta$ arise. They are linear in **A** in the Ginzburg-Landau functional whose Eq. (9) is a first variation. Because of the Coulomb gauge, terms of the kind ${\mathscr{A}}\cdot \nabla \eta$ give a null contribution to the functional and consequently should not appear in the corresponding GL equation. The remaining term is simply

52 $${\eta }^{\ast }\nabla \eta -\eta \nabla {\eta }^{\ast }.$$

Since $\eta$ is complex, it can be equivalently rephrased as

53 $${\eta }^{\ast }\nabla \eta -\eta \nabla {\eta }^{\ast }=2i({\rm{Im}}\,\eta \nabla {\rm{Re}}\,\eta -{\rm{Im}}\,\eta \nabla {\rm{Re}}\,\eta \mathrm{)}.$$

The last approximation we perform, together with the HFBP scheme and Eq. (49), is the statistical independence of the real and imaginary part of $\eta$. It can be shown that, up to the Gaussian order in the fluctuating fields, within the usual perturbative scheme^4^, this assumption holds. In the end, no contribution comes from the right-hand-side of Eq. (9), therefore one is left with the following equation for ${\mathscr{A}}$

54 $$\left[2\gamma {({e}^{\ast })}^{2}({\psi }_{0}^{2}+\langle |\eta {|}^{2}\rangle )+\frac{1}{{\mu }_{0}}\nabla \wedge \nabla \wedge \right]{\mathscr{A}}\mathrm{=0}.$$

In the end, within the HFBP we have derived three GL equations, one for the homogeneous background ${\psi }_{0}$, Eq. (17), the other two for $\eta$ and ${\mathscr{A}}$, respectively Eqs. (50) and (54).

### Including the BKT contribution

As a starting point, we take the field $\psi ({\bf{r}})$ in its phase-amplitude representation given by Eq. (41). In order to compute the additional BKT shift, as a first approximation^15,20^ one can consider the following phase-only functional

55 $$ {\mathcal F} [\phi ]={ {\mathcal F} }_{0}+\frac{J(T)}{2}\,\int \,{d}^{2}{\bf{r}}|\nabla \phi ({\bf{r}}{)|}^{2}\mathrm{}.$$

The phase stiffness $J(T)$ is defined in terms of the original GL parameters, i.e.

56 $$J(T)=\frac{2\gamma \alpha }{b}{k}_{B}(T-{T}_{c}).$$

The key point in the Eqs. (55) and (56) consists in the *benchmark* temperature in $J(T)$: it is no more ${T}_{c0}$ as for $a(T)$ in Eq. (4), but the fluctuation-shifted ${T}_{c}$ in Eq. (40).

As already clear from the seminal papers^24–26^, the major step forward in the BKT understanding was the fact that it is actually a topological transition. Indeed, the compactness of the phase field $\phi ({\bf{r}})$ implies the possibility for the system to display excited configurations (compared to the uniform one) which cannot be reached by continuously deforming the order parameter^4^. In $d=2$ these excitations are simply vortices and antivortices, depending on the sign of their (topological) charge $\nu \in {\rm{Z}}$, defined in terms of phase winding

57 $$\frac{1}{2\pi }\,{\oint }_{\Gamma }\,{d}^{2}{\bf{r}}\,\nabla \phi ({\bf{r}})=\nu \mathrm{}.$$

Now, a standard approach consists in drawing an electrostatic analogy and treating vortices and antivortices as a Coulomb gas moving in a uniform and neutral background^4,5,33^. The BKT critical point then divides two different phase of this vortex gas: for $T < {T}_{BKT}$, bound states of vortex-antivortex are predominant, while they are unbound above the critical temperature, destroying every global coherence property, such as superfluidity. Remarkably, this appears in a discontinuous way. Indeed, the transition displays a universal jump in the phase stiffness (and, consequently, in the superfluid density). This peculiar feature of the transition can be used to compute the critical temperature, according to the so-called Nelson-Kosterlitz criterion^26^

58 $${T}_{BKT}=\frac{\pi }{2}J({T}_{BKT})$$

reading Eq. (42) in the main text.
